# Comparison of the New-Generation Self-Expanding NAVITOR Transcatheter Heart Valve with Its Predecessor, the PORTICO, in Severe Native Aortic Valve Stenosis

**DOI:** 10.3390/jcm12123999

**Published:** 2023-06-12

**Authors:** Clemens Enno Eckel, Won-Keun Kim, Christina Grothusen, Vedat Tiyerili, Albrecht Elsässer, Dagmar Sötemann, Judith Schlüter, Yeong-Hoon Choi, Efstratios I. Charitos, Matthias Renker, Christian W. Hamm, Guido Dohmen, Helge Möllmann, Johannes Blumenstein

**Affiliations:** 1 Department of Cardiology, St. Johannes Hospital, 44137 Dortmund, Germany; clemens.eckel@joho-dortmund.de (C.E.E.); christina.grothusen@joho-dortmund.de (C.G.); vedat.tiyerili@joho-dortmund.de (V.T.); dagmar.soetemann@joho-dortmund.de (D.S.); judith.schlueter@joho-dortmund.de (J.S.); helge.moellmann@joho-dortmund.de (H.M.); 2 Department of Cardiology, University of Oldenburg, 26129 Oldenburg, Germany; elsaesser.albrecht@klinikum-oldenburg.de; 3 Department of Cardiac Surgery, Kerckhoff Heart Center, 61231 Bad Nauheim, Germany; w.kim@kerckhoff-klinik.de (W.-K.K.); y.choi@kerckhoff-klinik.de (Y.-H.C.); e.charitos@kerckhoff-klinik.de (E.I.C.); 4 Department of Cardiology, Kerckhoff Heart Center, 61231 Bad Nauheim, Germany; m.renker@kerckhoff-klinik.de; 5 Department of Cardiac and Vascular Surgery, University of Kiel, 24098 Kiel, Germany; 6 German Center for Cardiovascular Research (DZHK), Partner Site Rhine-Main, 55131 Bad Nauheim, Germany; c.hamm@kerckhoff-klinik.de; 7 Department of Cardiology, University of Giessen, 35390 Giessen, Germany; 8 Department of Cardiac Surgery, St. Johannes Hospital, 44137 Dortmund, Germany; guido.dohmen@joho-dortmund.de

**Keywords:** aortic stenosis, TAVI, TAVR, self-expanding prosthesis, paravalvular leak

## Abstract

Background: Third-generation transcatheter heart valves (THVs) are designed to improve outcomes. Data on the new intra-annular self-expanding NAVITOR are scarce. Aims: The aim of this analysis was to compare outcomes between the PORTICO and the NAVITOR systems. Methods: Data from 782 patients with severe native aortic stenosis treated with PORTICO (*n* = 645) or NAVITOR (*n* = 137) from 05/2012 to 09/2022 were evaluated. The clinical and hemodynamic outcomes of 276 patients (PORTICO, *n* = 139; NAVITOR, *n* = 137) were evaluated according to VARC-3 recommendations. Results: Rates of postprocedural more-than-mild paravalvular leakage (PVL) were significantly lower for NAVITOR than for PORTICO (7.2% vs. 1.5%, *p* = 0.041). In addition, severe bleeding rates (27.3% vs. 13.1%, *p* = 0.005) and major vascular complications (5.8% vs. 0.7%, *p* = 0.036) were lower in the NAVITOR group. The mean gradients (7 vs. 8 mmHg, *p* = 0.121) and calculated aortic valve areas (1.90 cm^2^ vs. 1.99 cm^2^, *p* = 0.235) were comparable. Rates of PPI were similarly high in both groups (15.3 vs. 21.6, *p* = 0.299). Conclusions: The NAVITOR demonstrated favorable in-hospital procedural outcome data, with lower rates of relevant PVL, major vascular complications, and severe bleeding than its predecessor the PORTICO and preserved favorable hemodynamic outcomes.

## 1. Introduction

The variety of transcatheter heart valves (THVs) for the treatment of severe aortic stenosis (AS) is continuously evolving. In the third generation, design adaptations have mainly addressed issues such as paravalvular leakage (PVL) and the occurrence of conduction disturbances leading to permanent pacemaker implantation (PPI). With increasing global experience and accumulating data, the impact of different valve designs on clinical outcomes is increasingly becoming apparent. Among self-expanding prostheses, the PORTICO and NAVITOR are characterized by an intra-annular position of the leaflets. Thus, they embody both the advantages of improved coronary access and hemodynamic properties of self-expandable prostheses. In the IDE clinical trial, PORTICO failed to demonstrate non-inferiority in direct comparison with the competitor devices: disadvantages of the PORTICO system were particularly evident in terms of PVL, major vascular complications, rates of PPI, and even mortality at 30 days [1]. In the course of improving the prosthesis, the rate of relevant PVL as well as vascular complications were particularly addressed. In January 2023, the FDA approved the NAVITOR for the American market. 

Data comparing the PORTICO and its direct successor, the NAVITOR, are not yet available. The aim of this analysis was to compare outcomes between the PORTICO and the NAVITOR. 

## 2. Methods

### 2.1. Patient Cohort

**Figure 1:**
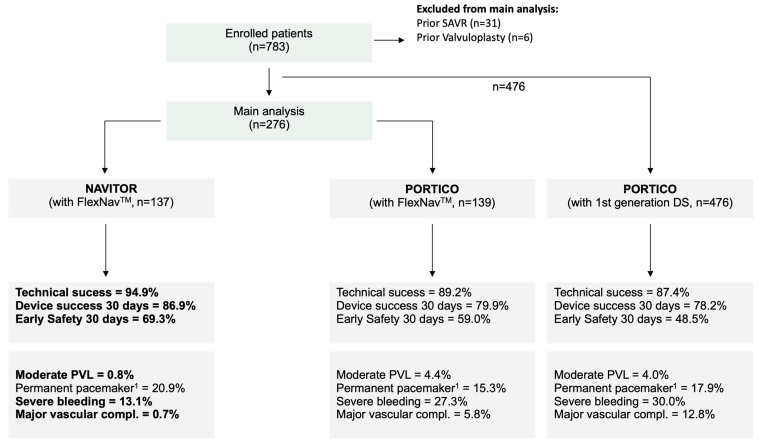
Study flowchart and procedural outcomes. Abbreviations: DS = delivery system; PVL = paravalvular leakage; compl. = complications. Bold outcomes for NAVITOR denote significant difference from values for PORTICO. ^1^ Excludes patients with permanent pacemaker at baseline.

**Figure 2:**
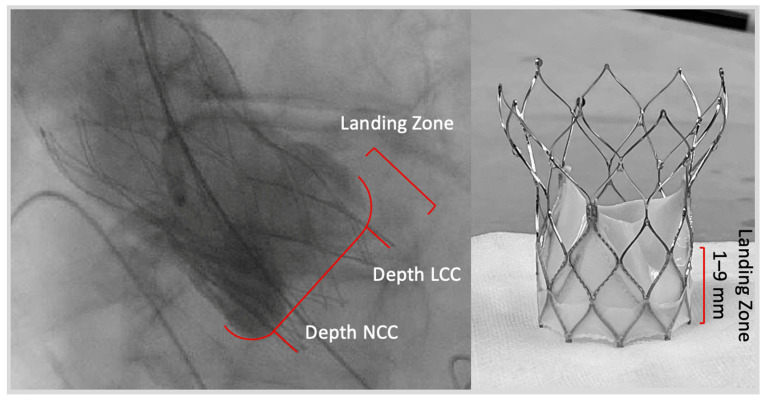
Landing zone and implantation depth. Abbreviations: LCC = left coronary cusp; NCC = non-coronary cusp.

The patient cohort comprised consecutive patients with symptomatic severe native AS who underwent transfemoral transcatheter aortic valve replacement (TAVR) between May 2012 and September 2022 using the PORTICO (*n* = 645) or NAVITOR (*n* = 137) (Abbott, Chicago, IL, USA). They were retrospectively included from two German high-volume centers (St. Johannes Hospital, Dortmund; Kerckhoff Heart Center, Bad Nauheim, Germany). Valve selection was performed by the local heart team for every individual patient. Due to lacking comparative data, data selection was based on local experience. Patients with type 0 native bicuspid valves were not evaluated for PORTICO as well as NAVITOR. After the exclusion of patients with the first-generation delivery system (*n* = 476), previous surgical aortic valve replacement (*n* = 31), and prior valvuloplasty (*n* = 6), the main cohort (*n* = 263) was grouped as to whether patients received the PORTICO (*n* = 139) or NAVITOR (*n* = 137) (Figure 1). Baseline characteristics such as comorbidities, risk scores, echocardiography and multidetector computed tomography (MDCT) results, and cardiac catheterization data were prospectively acquired in a dedicated database, as well as procedural data and complications. The implantation depth of the device was measured in the angiographic cusp-overlap view (see Figure 2). Follow-up data were collected at ambulatory visits, by telephone interview, or from recent medical reports. 

The study was conducted according to the Declaration of Helsinki. Due to the retrospective nature of the study and anonymous data processing, the need for approval by the respective local ethics committees was waived.

### 2.2. Multidetector Computed Tomography

MDCT was performed using dual-source technology (Somatom Definition or Somatom Force, Siemens Healthcare, Forchheim, Germany), as previously described [2]. Dedicated software was used for the analysis of the MDCT datasets (3mensio; version 1.2.5042, Pie Medical, Bilthoven, The Netherlands). In addition to standard measurements of the aortic root dimensions, the cover index (CI = 100 × (prosthesis diameter − perimeter-derived annulus diameter)/prosthesis diameter (%)), and the relationship between the sinotubular junction (STJ) and the perimeter-derived annulus (STJ-annulus index = 100 × (STJ − perimeter-derived annulus)/STJ (%)) were calculated. The aortic valve (AV) calcium score (AVCS) was measured according to the Agatston method using non-contrast-enhanced MDCT [3]. The calcium density was calculated as AVCS/annular area (AU/cm^2^) [4]. The presence of eccentric AV calcification and relevant left ventricular outflow tract (LVOT) calcification was determined by visual estimation of the AV in short-axis views and maximum intensity projections, as previously described [5]. 

### 2.3. Device Description

The PORTICO is available in 4 sizes (23, 25, 27, and 29 mm). The bovine pericardial leaflets as well as the porcine pericardial sealing sleeve are implemented in the self-expanding nitinol frame of the prosthesis. Due to the special retrieving mechanism, the prosthesis can be retracted into the delivery system and repositioned until it is 80% in place. Further technical features have already been described in detail [6]. 

The NAVITOR is the successor model to the PORTICO. After adaptation of the prosthesis, it now offers a new and especially active PVL sealing cuff (NaviSeal™) that fills and expands during diastole like a parachute [7].

A 14/15F sheathless delivery system (FlexNav™) became available for PORTICO during data acquisition and was used from 03/2020 in the present cohort. To allow a valid head-to-head comparison between PORTICO and NAVITOR, only patients treated with the new delivery system were included in the main analysis (Figure 1). 

The technical features of both THVs as well as their sheath dimensions and sizing recommendations are summarized in Supplementary Table S1. 

### 2.4. Outcomes 

The primary outcome measure was technical success according to VARC-3. Secondary outcome measures were 30-day all-cause mortality, device success at 30 days, and the early safety combined endpoint at 30 days [8]. 

### 2.5. Statistical Analysis

Statistical analysis was conducted using dedicated software (R version 4.2.1 (2021), R Foundation for Statistical Computing, Vienna, Austria). Continuous data are given as median and interquartile range (IQR) and categorical data as *n* (%). Comparison of the groups was accomplished using the Mann–Whitney U test and Fisher’s two-tailed exact test or the chi-squared test, as indicated. Mortality at 30 days was calculated by the Kaplan–Meier method and expressed by hazard ratios (HRs) and 95% confidence intervals (CIs). For all analyses, a two-sided *p*-value < 0.05 was considered significant. 

## 3. Results

### 3.1. Baseline Data

The final cohort consisted of 276 patients (PORTICO, *n* = 139; NAVITOR, *n* = 137). The mean age was 82.6 ± 5.6 years and 61.2% were female; further details are provided in Table 1. For the overall population see Supplementary Table S2. The population did not differ in baseline parameters.

### 3.2. Procedural Data and Outcomes 

Rates of pre-dilatation were comparable between the PORTICO and NAVITOR groups (91.4% vs. 90.4%, *p* = 0.939) (Table 2). The duration of the PORTICO implantation was significantly longer than that of the NAVITOR. The hemodynamic outcomes were comparable regarding mean gradients (7.0 [6.00; 9.00] mmHg vs. 8.0 [6.00; 10.50] mmHg, *p* = 0.121) and calculated aortic valve areas (1.90 [1.65; 2.11] mm^2^ vs. 1.99 [1.65; 2.20] mm^2^, *p* = 0.235). There was a lower rate of relevant PVL (7.2% vs. 1.5%, *p* = 0.041) in the NAVITOR group. In addition, a tendency towards greater technical success (89.2% vs. 94.9%, *p* = 0.128), early safety at 30 days (59.0% vs. 69.3%, *p* = 0.096), and device success at 30 days (79.9% vs. 86.9%, *p* = 0.162) was observed. The use of prosthesis sizes varied between PORTICO and NAVITOR (23 mm: 3.6% vs. 7.3%; 25 mm: 27.3% vs. 25.5%; 27 mm: 33.1% vs. 46.0%; 29 mm: 36.0% vs. 21.2%). The rate of associated PPI was high in both groups, without significant differences (15.3 vs. 21.6, *p* = 0.299). Further procedural characteristics and in-hospital events for the study cohort are provided in Figure 1 and Table 2, and Supplementary Table S3 shows data for the overall population.

### 3.3. Outcome Analysis up to 30 Days

**Figure 3:**
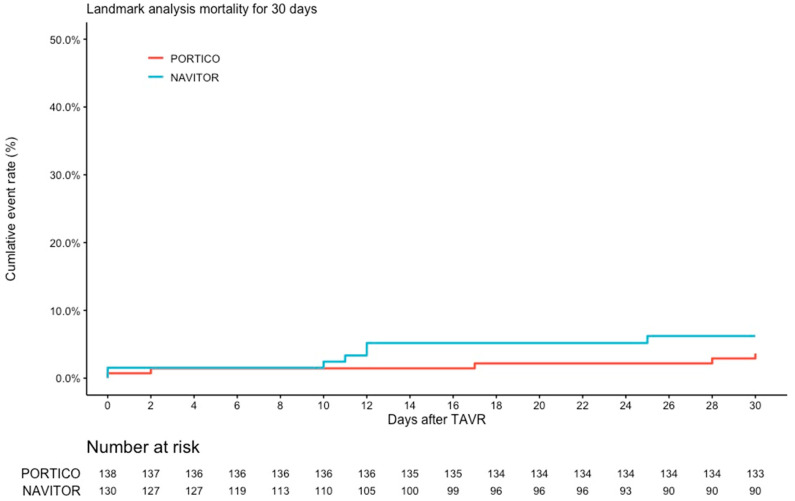
Kaplan–Meier curves for mortality up to 30 days ^1^. Annotation: ^1^ Lost to follow up at 30 days: *n* = 33 (12.0%); 8 patients deleted due to missing data.

The rate of in-hospital death did not differ between the two THVs (2.2% vs. 3.7%; *p* = 0.497). A closer look into the causes of death in the NAVITOR subgroup revealed two procedural deaths (due to device embolization), one cardiac death (due to decompensated mitral regurgitation), and two non-cardiac deaths (septic/inflammatory). In the PORTICO subgroup there was one death due to a major vascular complication with concomitant severe bleeding, two deaths due to device embolization, and one non-cardiac death (severe gastrointestinal bleeding). There were no significant differences regarding all-cause mortality (3.6% vs. 6.2%, HR 1.8; 95% CI 0.57–5.56; *p* = 0.335) up to 30 days (Figure 3). The incidence of permanent pacemaker use was comparable between the two groups (15.3% vs. 20.9%, *p* = 0.271). 

## 4. Discussion

This is the first head-to-head comparison between the PORTICO and its direct successor, the NAVITOR. Our main findings are: (1) the rate of more-than-mild PVL was lower with the NAVITOR without an increase in PPI rate; (2) the NAVITOR demonstrated favorable in-hospital procedural outcomes; (3) there was no difference regarding in-hospital and 30-day mortality; (4) vascular complications were less frequent with the NAVITOR.

As the periprocedural workflow has become more standardized, the current success rate of TAVR is high [9]. Across generations of prostheses, typical TAVR complications have decreased dramatically. In particular, the disadvantages of PVL, PPI, difficult coronary access, and peri-interventional stroke often attributed to the transcatheter procedure continue to play a smaller role, making the procedure appropriate also for low-risk collectives. The comparative analysis of different prosthesis systems and generations is therefore of particular importance for an optimized differential selection of prostheses in clinical practice [10]. The PORTICO-IDE trial investigated the PORTICO versus a variety of other, commercially available, valves (Edwards SAPIEN 3, CoreValve EVOLUT R/Pro). Non-inferiority of the PORTICO was not demonstrated, as mortality and the rates of PVL and PPI were higher compared with the competitors. However, in a post hoc analysis, no superiority was achieved for either the Edwards SAPIEN or Medtronic CoreValve prostheses [1]. Clinical outcome data on the NAVITOR, the direct and especially improved successor to the PORTICO, are scarce to date, and new data might affect the results of THV comparisons in the future.

### 4.1. Procedural Outcome 

Access-related complications and major bleeding are known to be closely associated with unfavorable outcomes [11,12,13,14]. Although both valve types were implanted using the same delivery system (FlexNav^TM^), major vascular complications and bleeding events were less frequent using the NAVITOR. However, this might be rather attributed to a learning curve and increasing general experience, as an impact of the valve design on vascular complications is unlikely. Due to updated VARC-3 criteria, a direct comparison with prior studies is difficult. However, incidences below 1% for major vascular complications and 12.9% for major bleedings, as defined by VARC-3, are remarkably low compared with prior studies [15]. Although it cannot be clearly proven with figures, clinical experience shows a more stable positioning of the device and its delivery system. The improved implantation technique could, therefore, also be reflected in the form of faster learning curve effects in the future.

### 4.2. Hemodynamic Outcome

The NAVITOR revealed low postprocedural gradients (8.0 mmHg) accompanied by a large AV area (1.99 cm^2^) and low rates of severe prosthesis–patient mismatch [16]. Despite the intra-annular design, which is often considered to be hemodynamically disadvantageous, the data are comparable to supra-annular self-expanding prostheses such as the ACURATE *neo2* (7.9 mmHg; 1.7 cm^2^) and the CoreValve Evolut *Pro* (6.4 mmHg; 2.0 cm^2^) [17,18,19,20]. Whether these results might also be expected in very small annuli (<400 cm^2^), as previously shown for Acurate neo, is still unknown and needs to be addressed in further trials [21]. Higher-grade residual PVL after TAVR is usually associated with an unfavorable outcome due to the long-term volume load of the left ventricle [1,22]. Because of its impact on the long-term outcome, third-generation devices have been adapted in the landing zone to further mitigate the incidence of residual PVL. As the main difference to the PORTICO design, the NAVITOR provides a skirt—the *NaviSeal*—that allows filling in the diastole, thus adapting to the calcified anatomy. Studies thus far have described a clear advantage in terms of the incidence of PVL with the *NaviSeal* [7]. Our data confirm a lower rate of relevant PVL (1.5%) after NAVITOR implantation than observed with the PORTICO (7.2%). In addition, the 30-day rate of moderate PVL after NAVITOR implantation (moderate: 0.0%) is comparable to that of other self-expanding prostheses, including ACURATE *neo2* (moderate: 3.0%) and the CoreValve EVOLUT *Pro* (moderate: 0.0%) [7,18,20]. Whether these low rates of PVL are also confirmed in the low- and intermediate-risk patient population will be shown by the data from the currently ongoing Vantage study.

### 4.3. Conduction Disturbances and Permanent Pacemaker Implantation 

PPI after TAVR has been identified as an isolated predictor of mortality [23]. However, any modification or enlargement of the stent profile by additional material such as a skirt could affect the incidence of severe conduction disturbances. In line with prior data of the CONFICENDE registry, our study revealed a similarly high PPI rate in both THV groups [15]. In addition to the amount of native valve calcium and preexisting right bunch bundle block, the implantation depth of the prothesis was identified as an isolated predictor of the need for PPI after TAVR [24]. Techniques to reduce the risk of conductance disturbances include the use of the cusp-overlap view instead of the standard 3-cusp view, mainly during implantation of self-expanding but also balloon-expandable prostheses [25,26,27]. The main reason for this important finding is presumably due to a more precise valve implantation relative to the conduction system by visually elongating the LVOT and accentuating the right non-commissure in the center of the fluoroscopic view. This technique was not used in the present cohort but has been applied to standard of care since then and will most likely further reduce the PPI rate and also ease access to the coronary arteries [28,29]. 

### 4.4. Limitations

The present analysis is limited by its retrospective, non-randomized nature. The relatively long period over which the study was conducted also introduces bias due to learning curves and different procedural approaches (e.g., changes in pre/post-dilatation strategies, radial access for pigtail catheter). There was no adverse event monitoring, and imaging data were not analyzed by a core laboratory. LVOT calcification and eccentric AV calcification were assessed visually without further quantification. 

## 5. Conclusions

In this first comparison with its predecessor the PORTICO, the NAVITOR demonstrated favorable in-hospital procedural outcome data: it had lower rates of relevant PVL, major vascular complications, and severe bleeding, with preserved favorable hemodynamic outcomes. Nevertheless, the incidence of PPI remains high after NAVITOR, which might be reduced by implementing the cusp-overlap technique during implantation. 

## Figures and Tables

**Table 1:** Baseline characteristics of the main study cohort ^1^.

Variable	PORTICO	NAVITOR	*p* Value
	*n* = 139	*n* = 137	
Demographic Data			
Age, years	82.7 [80.0; 86.0]	83.0 [80.0; 86.0]	0.382
Female sex	85 (61.2%)	84 (61.3%)	1.000
BMI, kg/m^2^	26.0 [23.1; 29.7]	26.9 [24.0; 30.1]	0.070
EuroSCORE I, %	13.9 [9.5; 22.4]	12.1 [8.4; 19.7]	0.141
EuroSCORE II, %	3.7 [2.2; 6.2]	3.6 [2.1; 5.1]	0.234
eGFR, mL/min/1.73 m^2^	56.0 [40.0; 71.5]	52.0 [38.0; 71.0]	0.412
Peripheral artery disease	32 (23.0%)	23 (16.8%)	0.252
Prior stroke	16 (11.5%)	12 (8.8%)	0.591
Atrial fibrillation	48 (34.5%)	56 (40.9%)	0.335
Coronary artery disease	90 (64.7%)	94 (68.6%)	0.580
Prior coronary intervention	54 (38.8%)	51 (37.2%)	0.878
Echocardiographic data			
LV ejection fraction, %	60.0 [51.0; 65.0]	60.0 [53.0; 65.0]	0.508
Mean gradient, mmHg	41.0 [29.5; 49.5]	41.0 [32.0; 49.0]	0.730
AVA, cm^2^	0.7 [0.6; 0.9]	0.8 [0.6; 0.9]	0.527
Electrocardiographic data			
Right bundle branch block	12 (9.0%)	9 (6.7%)	0.637
Left bundle branch block	5 (3.7%)	9 (6.7%)	0.418
Atrioventricular block	20 (15.0%)	24 (17.8%)	0.660
MDCT data			
Annular area, cm^2^	4.5 [4.0; 4.9]	4.4 [3.9; 4.8]	0.098
Annulus diameter, mm	24.3 [22.9; 25.8]	24.0 [22.6; 25.0]	0.061
LVOT, mm	24.0 [22.4; 25.6]	23.4 [22.1; 25.6]	0.252
STJ, mm	28.4 [26.6; 30.3]	28.1 [26.0; 29.9]	0.177
Aortic valve calcification, AU	2328 [1464; 3239]	2124 [1342; 3358]	0.556
Calcium density, AU/cm^2^	271 [108; 571]	301 [117; 630]	0.452

Data represent *n* (%) or median [interquartile range]. Abbreviations: BMI = body mass index; eGFR = estimated glomerular filtration rate; AVA = aortic valve area; MDCT = multidetector computed tomography; LVOT = left ventricular outflow tract; STJ = sinotubular junction; LV = left ventricle. ^1^ Excludes patients with first-generation delivery system (*n* = 476).

**Table 2:** Procedural outcomes and complications of the main study cohort ^1^.

Variable	PORTICO	NAVITOR	*p* Value
	*n* = 139	*n* = 137	
Procedural parameter			
Procedural duration, min	50.0 [40.0; 60.0]	45.0 [40.0; 55.0]	0.016
Contrast agent, mL	127.0 [98.0; 158.5]	120.0 [99.5; 161.5]	0.864
Pre-dilatation, %	127 (91.4%)	122 (90.4%)	0.939
Post-dilatation, %	48 (35.6%)	33 (25.0%)	0.081
Depth NCC, mm	4.0 [3.0; 6.0]	4.00 [2.0; 5.0]	0.052
Depth LCC, mm	4.0 [2.0; 5.0]	3.0 [1.0; 5.0]	0.088
Echocardiographic outcome			
LV ejection fraction, %	60.5 [53.0; 65.0]	60.0 [54.0; 65.0]	0.859
Mean gradient, mmHg	7.0 [6.0; 9.0]	8.0 [6.0; 10.5]	0.121
AVA, cm^2^	1.9 [1.7; 2.1]	2.0 [1.7; 2.2]	0.235
Relevant PVL (>mild/trace or SAVR/ViV due to PVL)	10 (7.2%)	2 (1.5%)	0.041
Severe PPM	3 (2.7%)	1 (0.8%)	0.353
Clinical and procedural outcome			
Technical success	124 (89.2%)	130 (94.9%)	0.128
Device success at 30 days	111 (79.9%)	119 (86.9%)	0.162
Early safety at 30 days	82 (59.0%)	95 (69.3%)	0.096
In-hospital death	3 (2.2%)	5 (3.7%)	0.497
Periprocedural death (in-hospital and up to 30 days)	6 (4.3%)	7 (5.2%)	0.968
Conversion to sternotomy	0 (0.0%)	1 (0.7%)	0.496
Multiple valves (ViV)	5 (3.6%)	2 (1.5%)	0.447
Device migration/embolization	8 (5.8%)	3 (2.2%)	0.218
Major vascular complication	8 (5.8%)	1 (0.7%)	0.036
Severe bleeding (type 2–4)	38 (27.3%)	18 (13.1%)	0.005
Major cardiac structural complication	4 (2.9%)	4 (2.9%)	1.000
All stroke (overt CNS injury)	5 (3.6%)	3 (2.2%)	0.723
AKI (type 2–4)	5 (3.6%)	4 (2.9%)	1.000
New permanent pacemaker ^2^	19 (15.3%)	24 (20.9%)	0.271

Data represent *n* (%) or median [interquartile range]. Abbreviations: LCC = left coronary cusp; NCC = non-coronary cusp; AVA = aortic valve area; PVL = paravalvular leakage; CNS = central nervous system; PPM = prosthesis–patient mismatch; SAVR = surgical aortic valve replacement; ViV = valve-in-valve; AKI = acute kidney injury. ^1^ Excludes patients with the older delivery system (*n* = 476). ^2^ Excludes patients with pacemaker at baseline (*n* = 37).

## Data Availability

Data is contained within the article or Supplementary Material.

## Supplementary Materials

The following supporting information can be downloaded at: https://www.mdpi.com/article/10.3390/jcm12123999/s1, Table S1. Sizing recommendations for PORTICO and NAVITOR. Table S2. Baseline characteristics of the overall population. Table S3. Procedural outcomes and complications (overall population).

## Abbreviations

AV	aortic valve
CI	cover index
LVOT	left ventricular outflow tract
MDCT	multidetector computed tomography
PPI	permanent pacemaker implantation
PVL	paravalvular leakage
MDCT	multidetector computed tomography
STJ	sinotubular junction
TAVR	transcatheter aortic valve replacement
THV	transcatheter heart valve
VARC	Valve Academic Research Consortium
