# Supplementary material for: Comparison of the New-Generation Self-Expanding NAVITOR Transcatheter Heart Valve with Its Predecessor, the PORTICO, in Severe Native Aortic Valve Stenosis
Source: J Clin Med. 2023 Jun 12;12(12):3999. doi: 10.3390/jcm12123999 (PMC10299266; doi:10.3390/jcm12123999)
Supplement: Supplementary file 1 [file jcm-12-03999-s001.zip › jcm-2397770-supplementary.pdf]

Supplementary Table 1. Sizing recommendations for PORTICO and NAVITOR.

	PORTICO				NAVITOR			
**Prosthesis parameters**								
Sizes, mm	23	25	27	29	23	25	27	29
**Recommended annulus ranges**								
Area, mm^2	277-346	338–415	405–491	479–573	277–346	338–415	405–491	479–573
Perimeter, mm	60-66	66-73	72-79	79-85	60-66	66-73	72-79	79-85
Diameter, mm	18.9-22.1	22.1-23.1	22.9-25.0	25.0-26.9	19-21	21-23	23-25	25-27
***FlexNav*™ Delivery System**								
14 F	14 F				15F			
Outer diameter	6.0 mm				6.3 mm			
Vessel diameter	≥5.0 mm				≥5.5 mm			

Supplementary Table 2. Baseline characteristics of the overall population.

Variable	**PORTICO** ***n=615***	**NAVITOR** **n=137**	**p Value**
Demographic data			
Age, years	82.9 [79.8;86.0]	83.0 [80.0;86.0]	0.375
Female sex, %	389 (63.3%)	84 (61.3%)	0.744
BMI, kg/m^2	26.6 [23.9;30.4]	26.9 [24.0;30.1]	0.517
EuroSCORE I, %	15.9 [10.2;23.7]	12.1 [8.4;19.7]	0.001
EuroSCORE II, %	3.4 [2.3;5.7]	3.6 [2.1;5.1]	0.479
eGFR, ml/min/1.73 m^2	56.0 [41.5;76.0]	52.0 [38.0;71.0]	0.071
Peripheral artery disease	103 (16.7%)	23 (16.8%)	1.000
Prior stroke	60 (9.8%)	12 (8.8%)	0.862
Atrial fibrillation	230 (37.4%)	56 (40.9%)	0.509
Coronary artery disease	329 (53.5%)	94 (68.6%)	0.002
Prior coronary intervention	221 (35.9%)	51 (37.2%)	0.852
**Echocardiographic data**			
LV Ejection fraction, %	60.0 [53.0;65.0]	60.0 [53.0;65.0]	0.560
Mean gradient, mmHg	42.0 [33.0;51.0]	41.0 [32.0;49.0]	0.180
AVA, cm^2	0.7 [0.6;0.8]	0.8 [0.6;0.9]	0.031
**Electrocardiographic data**			
Right bundle branch block	37 (6.1%)	9 (6.7%)	0.959
Left bundle branch block	51 (8.4%)	9 (6.7%)	0.621
Atrioventricular block	85 (14.0%)	24 (17.8%)	0.328
**MDCT data**			
Annular area, cm^2	4.4 [3.9;4.9]	4.4 [3.9;4.8]	0.087
Annulus diameter, mm	24.3 [22.9;25.6]	24.0 [22.6;25.0]	0.020
LVOT, mm	23.8 [21.9;25.5]	23.4 [22.1;25.6]	0.694
STJ, mm	28.3 [26.4;30.1]	28.1 [26.0;29.9]	0.471
Aortic valve calcification, AU	2328 [1636;3308]	2124 [1342;3358]	0.112
Calcium density, AU/cm^2	435 [168;667]	301 [117;630]	0.054

Data represent n (%) or median [interquartile range].
Abbreviations: BMI = body mass index; eGFR = estimated glomerular filtration rate; AVA = aortic valve area; LVOT = left ventricular outflow tract; STJ = sinotubular junction; LV = left ventricle.

Supplementary Table 3. Procedural outcomes and complications (overall population).

Variable	PORTICO	NAVITOR	p Value
	n=615	*n=137*	
**Procedural parameter**			
Procedural duration, min	50.00 [40.00;65.00]	45.00 [40.00;55.00]	0.003
Contrast agent, ml	120.00 [97.00;154.50]	120.0 [99.50;161.50]	0.981
Pre-dilatation, %	533 (86.66%)	122 (90.37%)	0.304
Post-dilatation, %	154 (25.21%)	33 (25.00%)	1.000
Depth NCC, mm	5.00 [3.00;6.00]	4.00 [2.00;5.00]	0.001
Depth LCC, mm	4.00 [2.00;6.00]	3.00 [1.00;5.00]	<0.001
**Echocardiographic outcome**			
LV ejection fraction, %	61 [55;65]	60 [54;65]	0.604
Mean gradient, mmHg	8.00 [6.00;10.00]	8.00 [6.00;10.50]	0.756
AVA, cm^2	0.95 [0.83;1.09]	1.08 [0.91;1.22]	<0.001
Relevant PVL (> mild/trace or SAVR/ViV due to PVL)	35 (5.69%)	2 (1.46%)	0.064
Severe PPM	17 (3.90%)	1 (0.84%)	0.141
**Clinical and procedural outcome**			
Technical success	540 (87.80%)	130 (94.89%)	0.024
Device success at 30 days	483 (78.53%)	119 (86.86%)	0.037
Early safety at 30 days	313 (50.89%)	95 (69.34%)	<0.001
In-hospital death	18 (2.92%)	5 (3.67%)	0.588
Periprocedural death (in-hospital and up to 30 days)	26 (4.22%)	7 (5.14%)	0.809
Conversion to sternotomy	6 (0.97%)	1 (0.73%)	1.000
Multiple valves (ViV)	18 (2.92%)	2 (1.46%)	0.555
Device migration / embolization	19 (3.10%)	3 (2.19%)	0.781
Major vascular complication	69 (11.22%)	1 (0.73%)	<0.001
Severe bleeding (type 2-4)	181 (29.43%)	18 (13.13%)	<0.001
Major cardiac structural complication	13 (2.11%)	4 (2.92%)	0.529
All stroke (overt CNS injury)	21 (3.41%)	3 (2.19%)	0.597
AKI (type 2-4)	28 (4.55%)	4 (2.92%)	0.534
New permanent pacemaker[1]	104 (20.00%)	24 (20.87%)	0.823

Data represent n (%) or median [interquartile range].
Abbreviations: LCC = left coronary cusp; NCC = non coronary cusp; AVA = aortic valve area; PVL = paravalvular leakage; CNS = central nervous system; SAVR = surgical aortic valve replacement; ViV = valve-in-valve; PPM = prosthesis-patient mismatch; AKI = acute kidney injury; LV = left ventricle.
[1]Excluded patients with pacemaker at baseline (n=117).
